# First-Principles Calculate the Stability, Mechanical Properties and Electronic Structure of Carbide MC, M_2_C and M_6_C in M50NiL Steel

**DOI:** 10.3390/ma17143498

**Published:** 2024-07-15

**Authors:** Xi Yong, Xiating Liu, Maosheng Yang, Xiaolong Zhou

**Affiliations:** 1 Institute for Special Steels, Central Iron and Steel Research Institute, Beijing 100081, China; yongxi@nercast.com; 2 Faculty of Materials Science and Engineering, Kunming University of Science and Technology, Kunming 650093, China; 20212230057@stu.kust.edu.cn

**Keywords:** carbides, stability, mechanical properties, electronic structure, first-principles calculations

## Abstract

In this paper, the stability, mechanical properties and electronic structure of carbides in steel were calculated using the first-principles method based on the density functional theory (DFT). Firstly, the MC, M_2_C, M_6_C (M = Cr, Mo, V, Fe) carbides models were established. Then, different interphases’ lattice constants, formation enthalpy, binding energy and elastic modulus were calculated. The stability, hardness, ductility and anisotropy of each phase were finally analyzed. The results show that these phases are stable, and the stability is closely related to the electron loss ability of its metal elements. The stronger the electron loss ability of its metal elements, the more stable the formed phase. As for MC carbides, MoC has the largest bulk modulus and hardness. As for M_2_C carbides, the Poisson’s ratio of Cr_2_C is the smallest, and all phases except for Cr_2_C show toughness and ductility. The anisotropy of M_6_C carbides is relatively poor.

## 1. Introduction

The research of bearing steel has lasted for more than a century to meet different mechanical properties under various service conditions [1]. M50NiL-bearing steel has excellent mechanical properties at high temperatures, so it is widely used in aerospace. M50NiL steel has high toughness in the core area after carburizing, which can meet the requirements of stable operation under the condition of a DN value of 3, and it is the most widely used bearing steel [2,3,4,5,6,7]. M50NiL steel shows high strength, hardness and wear resistance during use [8,9]. These excellent properties are closely related to the carbides formed by their internal elements. The main alloying elements in M50NiL steel are shown in Table 1. The main alloying elements are Cr, Mo, Ni, and V. Except for Ni, other alloying elements are easy to form carbides such as MC, M_2_C and M_6_C with C element [10,11,12], and these carbides’ structures and chemical compositions are different. It is precisely because of the formation of these carbides in bearing steel that makes the bearing steel have excellent properties. This has attracted the attention of researchers. A variety of different research methods were used for experimental and theoretical research. However, conducting experimental analysis on numerous carbides will significantly increase the material development cycle. With the development of computational materials science, using first-principles calculation can significantly reduce the material development cycle and be of great help to predict the properties of carbides.

Currently, numerous researchers have utilized experimental design to examine the properties of carbides in steel. It is mainly achieved by adjusting the material composition ratio, altering the structure and composition of phases in bearing steel, optimizing the characteristics, and ultimately enhancing the properties of bearing steel [3,14,15,16]. However, the low efficiency and high cost of research and development through experimental methods can no longer meet the design requirements for modern materials. The first-principles method has become a vital calculation method for modern material design. It studies the crystal structure of materials and combines the algorithm of quantum mechanics to understand the relationship between macroscopic property and microstructure of materials. Many scholars have currently used the first-principles method to study the structure and property of carbides. Guo et al. [17] studied the stability of MC and M_2_C carbides in bearing steel. During the heat treatment at 1200 °C, M_2_C carbides gradually transform into MC carbides until M_2_C carbides completely disappear. Jang et al. [18] studied the stability of (Ti, M)C and M(C, Va) (M = Nb, V, Mo, W) carbides with a NaCl crystal structure using the first-principles method. After Mo and W replace Ti atoms, the formation enthalpy values significantly increase, and the stability decreases. Wang et al. [19] studied the structure, stability and elastic properties of di-transition-metal carbides Ti_x_V_1−x_C by using the first-principles with a pseudopotential plane-waves method. The results show that the elastic properties of Ti_x_V_1−x_C (0 ≤ x ≤ 1) are varied by doping with V. The bulk modulus of Ti_0.5_V_0.5_C is larger than that of pure TiC, while Ti_0.5_V_0.5_C presents brittleness based on the analysis of ductile/brittle behavior. The Ti_0.5_V_0.5_C carbide has the lowest formation energy, indicating that Ti_0.5_V_0.5_C is more stable than all other alloys. According to the type of 3*d* transition metal, Suetin et al. [20] studied the structural, electronic and magnetic properties of η-carbides M_3_W_3_C (M = Ti, V, Cr, Mn, Fe, Co, Ni) using the first-principles calculations. The mechanical properties, such as elastic moduli, mechanical stability, ductility, and formation enthalpy were predicted and compared with those for binary M_x_C_y_ carbides. The volume modulus, shear modulus and Young’s modulus have parabolic dependences on 3D transition metal with the maximum values for Fe_3_W_3_C. Lv et al. [21] calculated the effect of atomic sites on the electronic and mechanical properties of (Fe, Mo)_6_C carbides by using first-principles calculations and found that the phase stability of Fe_3_Mo_3_C-II (Mo at the 48f site) is higher than other phases.

This paper studied the stability and mechanical properties of MC, M_2_C and M_6_C carbides in M50NiL steel by first-principles which are based on density functional theory. Through the existing experiment and calculation data [22], and according to each phase’s crystal system and lattice parameters, the models were established and calculated. Using first-principles calculations can not only significantly save experimental resources, but also can provide theoretical support for developing bearing steel with different mechanical properties under various service conditions.

## 2. Computational Details

**Figure 1:**
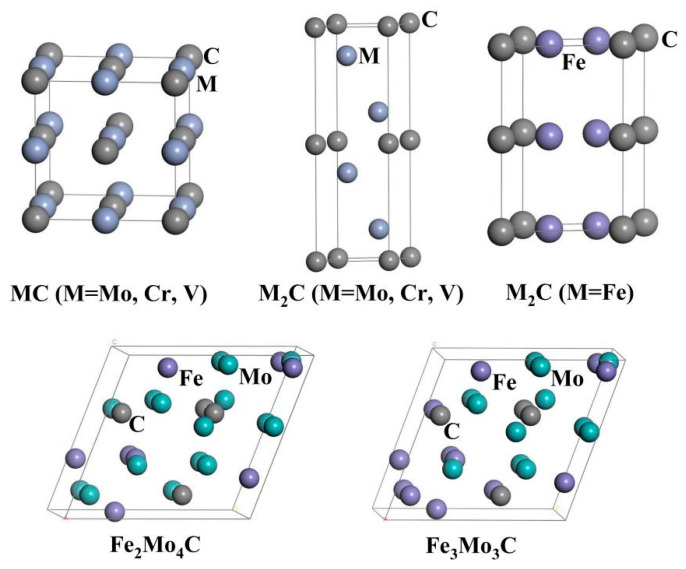
Computational models for different carbides.

The carbides in M50NiL steel are MC, M_2_C and M_6_C (M = Cr, Mo, V, Fe) types. The structural types and lattice parameters of these carbides are shown in Table 2. According to these parameters, different models are established using the first-principles method, as shown in Figure 1. The atoms are free to relax, and the unit cell parameters and atomic sites will change to achieve the most stable states. After the convergence test [23,24], GGA-PW91 is selected as the exchange-correlation function in the structural optimization process. The cutoff energy is 450 eV, the K-points is 9 × 9 × 9, the energy change is less than 1.0 × 10^−5^ eV/atom, the maximum pressure is 0.03 eV/Å, the internal stress is less than 0.02 GPa, the maximum displacement of atom is 0.001 Å, the number of self-consistent iterations is equal to 100, and the pseudopotential is ultrasoft pseudopotential.

The elastic constant *C*_ij_ describes the relationship between stress and strain during the deformation of materials with different structures [25]. After structural optimization, the elastic constants *C*_ij_ of different phases were calculated. The stress–strain relationship can be represented by a matrix [26]. For the triclinic crystal system, there are 21 independent matrix elements. The orthogonal crystal system has 21 independent matrix elements, while the cubic crystal system with the highest symmetry only has 3 matrix elements (*C*_11_, *C*_12_, *C*_44_). The steepness number used in this paper is 6, and the maximum strain amplitude is 0.005. The elastic constants were used to calculate the elastic moduli of different models, including the bulk modulus, shear modulus, Young’s modulus and Poisson’s ratio. The general anisotropy index $A^{U}$ was calculated, and the mechanical properties of different mesophases were analyzed. In this paper, MC, M_2_C and M_6_C carbides contained MoC [27], CrC [28], VC [29], Mo_2_C [30], Cr_2_C [31], V_2_C [32], Fe_2_C [10], Fe_2_Mo_4_C [33], Fe_3_Mo_3_C [34], respectively.

## 3. Results and Discussion

### 3.1. Crystal Parameters and Stability

The CASTEP module is used to optimize the geometry of carbides in this paper. Energies after structural optimization are shown in Table 3. The formation enthalpy and binding energy of different phases were calculated for the stability analysis of various intermediate phases. Formation enthalpy is the thermal effect of a stable element in a standard state at a specific temperature to produce a pure substance. The value of formation enthalpy can be used to determine the feasibility and difficulty of the reaction of the whole substance. When this value is negative, the smaller it is, the easier it is to carry out such a reaction. Binding energy is the energy that binds two or more parts of a system together. In general, the lower the binding energy of the crystal structure, the better the stability of crystal. Calculation formulas for formation enthalpy and binding energy are shown as follows [32]:

(1) $$H=E_{total}-xE_{solid}^{M}-yE_{solid}^{N}$$

(2) $$E_{xy}^{coh}=\frac{E_{total}-xE_{atom}^{M}-yE_{atom}^{N}}{x+y}$$

where *H* is the total formation enthalpy, and $E_{total}$ is the total energy of unit cell used in this calculation. $E_{solid}^{M}$ and $E_{solid}^{N}$ are the energy of single M atom and the energy of single N atom, respectively. The number of M atom and the number of N atom are represented by *x* and *y*, respectively. $E_{xy}^{coh}$ is the binding energy of crystal. $E_{atom}^{M}$ and $E_{atom}^{N}$ are the energy of free M atom and the energy of free N atom, respectively. Calculated formation enthalpy and binding energy values are concluded in Table 3.

**Figure 2:**
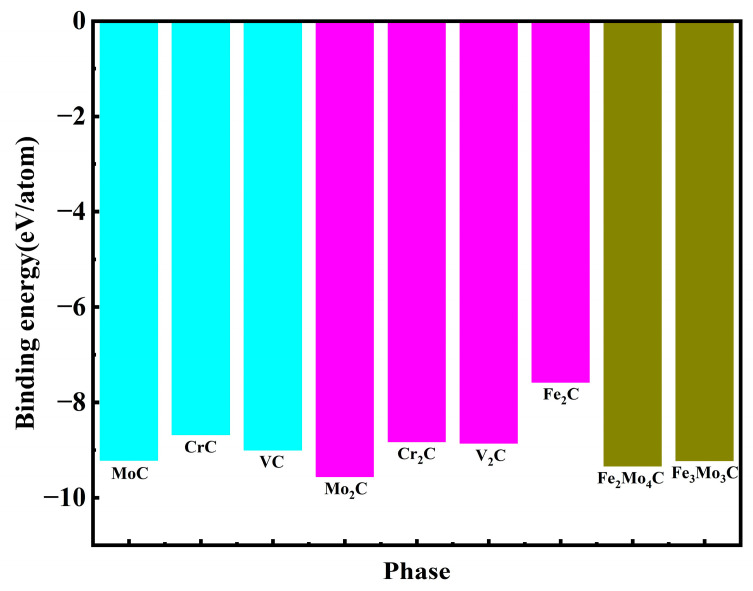
Binding energy of different carbides.

The binding energy was calculated to analyze the stability of each phase. The smaller the binding energy, the more stable the phase. As shown in Figure 2, the binding energies are all less than 0, indicating that these phases are stable. These phases have been experimentally proved to exist in M50NiL steel [30,31,33]. This also shows that these phases can be stable in steel. As we know, the value of binding energy is related to the structure and is also closely related to the metal formation elements. Concluded from Figure 2, the binding energy order of MC carbides is MoC < VC < CrC, which means that the stability order is MoC > VC > CrC. The binding energy order of M_2_C carbides is Mo_2_C < V_2_C < Cr_2_C < Fe_2_C, which indicates that the stability order is Mo_2_C > V_2_C > Cr_2_C > Fe_2_C. The binding energy order of M_6_C carbides is Fe_2_Mo_4_C < Fe_3_Mo_3_C, which indicates that Fe_2_Mo_4_C is more stable than Fe_3_Mo_3_C. In summary, it is found that for the second phase of the same type, the stronger the electron loss ability of its metal elements, the more stable the formed phase. The electron loss ability of these metal elements is Mo > V > Cr > Fe. As for M_2_C carbides, the stability order is Mo_2_C > V_2_C > Cr_2_C > Fe_2_C. As for M_6_C carbides, the stability order is Fe_2_Mo_4_C > Fe_3_Mo_3_C, and the reason lies in the content of Mo in Fe_2_Mo_4_C being greater than Fe_3_Mo_3_C. This is also consistent with the experimental results of Decaudin’s study [35]. In this study, the main element content and crystal structure of the main carbides in M50 steel were studied by extracting carbides from the steel and the EDX and STEM analyses. Table 4 shows the compositions of different carbides. The alloy content order is Mo > Cr > Fe in M_2_C carbides, indicating that the stability order is Mo_2_C > Cr_2_C > Fe_2_C. This is consistent with the calculation results. For M_6_C carbides, it can be seen from Table 4 that the content of Mo is greater than Fe, and the stability order is Fe_2_Mo_4_C > Fe_3_Mo_3_C. This is also consistent with the calculation results. In summary, the calculated stability order of various carbides is Mo_2_C > Fe_2_Mo_4_C > Fe_3_Mo_3_C > MoC > VC > V_2_C > Cr_2_C > CrC > Fe_2_C.

### 3.2. Mechanical Properties

The elastic constant *C*_ij_ and flexible constant *S*_ij_ of each phase were firstly calculated, and then the elastic modulus was calculated according to the following formulas. Elastic modulus is an important parameter to measure a material’s mechanical properties [36]. It mainly includes bulk modulus *B*, shear modulus *G*, Young’s modulus *E* and Poisson’s ratio *ν*. Lastly, the universal elastic anisotropy index $A^{U}$ was calculated. For different crystal systems, the number of elastic constants and the formulas for calculating the elastic modulus are both different. The bulk modulus and the shear modulus exhibit the material’s ability to resist deformation. The bulk modulus and the shear modulus of different crystal systems can be calculated as follows:

Cubic crystal system:

(3) $$B_{V}=B_{R}=\frac{1}{3}\left(C_{11}+2C_{12}\right)$$

(4) $$G_{V}=\frac{1}{5}\left(C_{11}-C_{12}+3C_{44}\right)$$

(5) $$G_{R}={\left[\frac{4}{5}{\left(C_{11}-C_{22}\right)}^{-1}+\frac{3}{5}C_{44}^{-1}\right]}^{-1}$$

Hexagonal crystal system:

(6) $$B_{V}=\frac{2\left(C_{11}+C_{12}\right)+C_{33}+4C_{13}}{9}$$

(7) $$G_{V}=\frac{C_{11}+C_{12}+2C_{33}-4C_{13}+12C_{44}+12C_{66}}{30}$$

(8) $$B_{R}=\frac{\left(C_{11}+C_{12}\right)C_{33}-2C_{13}^{2}}{30}$$

(9) $$G_{R}=\frac{5\left[\left(\left(C_{11}+C_{12}\right)C_{33}-2C_{13}^{2}\right)C_{44}C_{66}\right]}{2\left[3B_{V}C_{44}C_{66}+\left(\left(C_{11}+C_{22}\right)C_{33}-2C_{13}^{2}\right)\left(C_{44}+C_{66}\right)\right]}$$

Considering that the calculation results of the Voigt and Reuss models will be biased, Hill [37] took the arithmetic mean value of these two models’ calculation results to solve the deviation between the two calculated values and finally constructed the Hill model. The calculated formulas of bulk modulus (*B*) and shear modulus (*G*) are as follows [38,39]:

(10) $$B=\left(B_{R}+B_{V}\right)/2$$

(11) $$G=\frac{G_{R}+G_{V}}{2}$$

Young’s modulus (*E*) is used to assess the stiffness of the material, and the larger the Young’s modulus, the greater the stiffness of the material. Poisson’s ratio reflects the lateral deformation of the material and is often used to evaluate the shear resistance of the crystal structure of the material. The more significant the Poisson’s ratio, the better the plastic toughness of the material [35]. Young’s modulus and Poisson’s ratio can be calculated as follows [38]:

(12) $$E=\frac{9GB}{G+3B}$$

(13) $$\nu =\frac{3B-2G}{6B+2G}$$

Elastic anisotropy is also one of the most important mechanical characteristics of crystal materials [40]. It is related to the generation of microcracks in materials and the response of crystals under applied stress is closely related to anisotropy. The universal anisotropic index $A^{U}$ can describe the degree of anisotropy [41]. The calculation method is shown in the following formula. The magnitude of $A^{U}$ deviates from 0 and it represents the material’s elastic anisotropy degree. The greater the value deviates from 0, the greater the degree of anisotropy [42]. When $A^{U}$ = 0, it indicates that the material is isotropic [38]:

(14) $$A^{U}=5\frac{G_{V}}{G_{R}}+\frac{B_{V}}{B_{R}}-6$$

**Figure 3:**
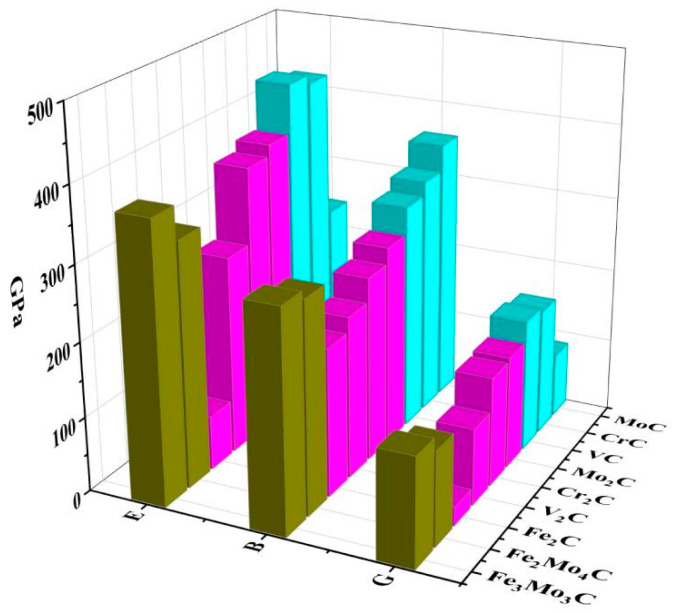
Comparison of *B*, *E* and *G* of different carbides.

The hardness of the material is closely related to the Young’s modulus and the shear modulus. In general, the larger the shear modulus and the Young’s modulus, the greater the hardness of material [43]. For convenient comparative analysis, Young’s modulus *E*, bulk modulus *B* and shear modulus *G* are drawn in Figure 3. It can be seen that carbides have a higher shear modulus and Young’s modulus than intermetallic compounds. It also indicates that carbides have better toughness and ductility. In particular, Young’s modulus represents the rigidity of material. The larger the Young’s modulus, the less likely the material will deform. As for MC carbides, the hardness order is VC > CrC > MoC. As for M_2_C carbides, the hardness order is Mo_2_C > Cr_2_C > V_2_C > Fe_2_C. While for the M_6_C carbides, the hardness order is Fe_3_Mo_3_C > Fe_2_Mo_4_C.

**Figure 4:**
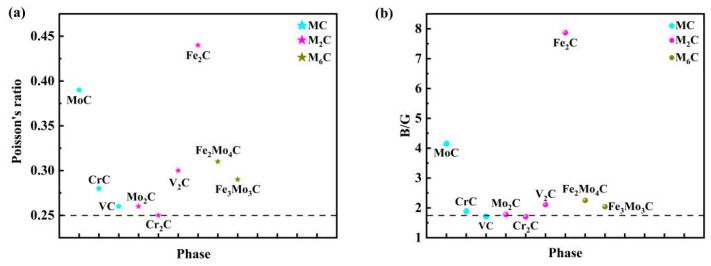
Comparison of Poisson’s ratio and *B*/*G* values of different carbides. (**a**) refers to the Poisson’s ratio value, and (**b**) refers to the B/G value. The value of dashed line in (**a**) corresponds to Poisson’s ratio = 0.25, and the value of dashed line in (**b**) corresponds to B/G = 1.75.

Poisson’s ratio reflects the transverse deformation of the material. Materials with a larger Poisson’s ratio show that the transverse deformation of the material is greater than the longitudinal deformation before the plastic deformation of the material with loaded force. It is often used to evaluate the shear resistance of the material crystal structure. Material with a large Poisson’s ratio shows better toughness and smaller hardness. The Poisson’s ratio of steel is generally around 0.3. As can be seen from Figure 4a, except for Cr_2_C, the toughness of other phases is greater than that of steel itself. The formation of these phases in steel can improve its toughness. The value of *B*/*G* can reflect the brittleness and toughness of the material. When it is lower than 1.75, it is brittle. When it is above 1.75, it indicates better toughness or ductility of the material. Figure 4b shows the *B/G* value of each phase. The *B*/*G* values of VC and Cr_2_C are less than 1.75, which shows greater brittleness. The remaining phases show good toughness. The formation of these phases in steel is beneficial to improve the toughness and ductility of M50NiL-bearing steel. As for the MC carbides, Poisson’s ratio and the *B*/*G* order are MoC > CrC > VC. As for the M_2_C carbides, Poisson’s ratio and the *B*/*G* order are Fe_2_C > V_2_C > Mo_2_C > Cr_2_C. As for the M_6_C carbides, Poisson’s ratio and the *B*/*G* order are Fe_2_Mo_4_C > Fe_3_Mo_3_C. Overall, the toughness and ductility order of all carbides is Fe_2_C > MoC > Fe_2_Mo_4_C > V_2_C > Fe_3_Mo_3_C > CrC > VC > Mo_2_C > Cr_2_C.

**Figure 5:**
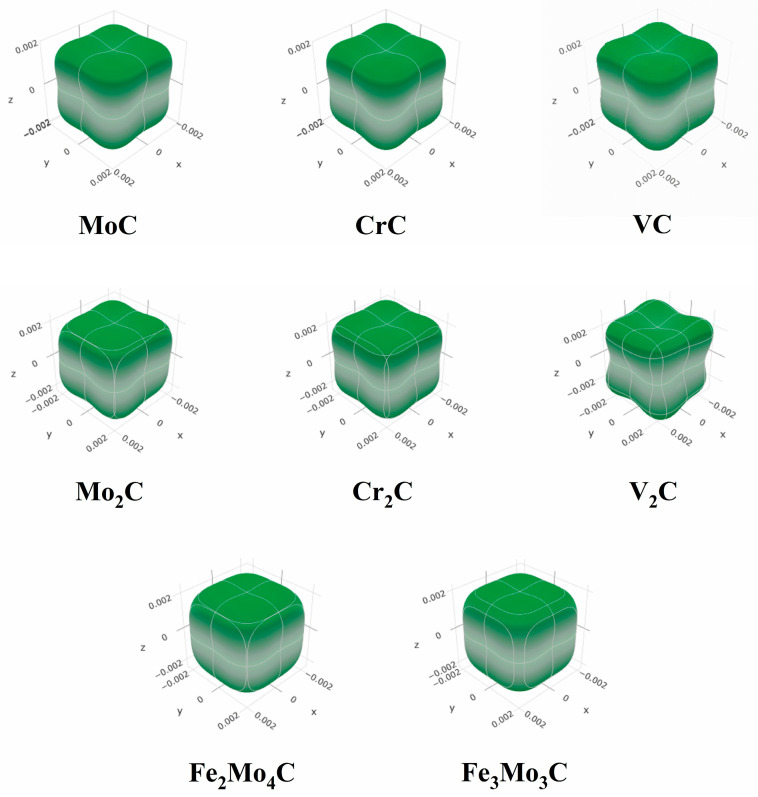
Spatial distribution of Young’s modulus.

The universal anisotropy index $A^{U}$ and Young’s modulus spatial distribution map can measure the degree of elastic anisotropy. Universal anisotropy index $A^{U}$ values are shown in Table 5. Based on the criteria described above, the anisotropy degree of different phases is MoC > V_2_C > VC > Cr_2_C > CrC > Mo_2_C > Fe_2_Mo_4_C > Fe_3_Mo_3_C. In order to deeply analyze the elastic anisotropy of each phase, the spatial distribution maps of Young’s modulus are drawn according to the flexibility coefficient *S*_ij_. Fe_2_C is too small to analyze the anisotropy due to its stiffness. Except for Fe_2_C, the rest of Young’s modulus spatial distribution maps are shown in Figure 5. The 3D graph of Young’s modulus should be perfectly spherical for the isotropic crystal, otherwise, the crystal is anisotropic [44]. It is found that the spatial distributions of Young’s modulus in all phases do not show the spherical shape; therefore, all the phases show anisotropy. The $A^{U}$ values of MoC, V_2_C, and CrC are relatively large, and the differences in the *x*, *y* and *z* directions are significant. The anisotropy of other phases decreases with the decreased $A^{U}$ value. The closer to 0 of the Young’s modulus spatial distribution, the smaller the difference in three directions. Fe_3_Mo_3_C and Fe_2_Mo_4_C show the slightest difference. According to the analysis of the spatial distribution of the $A^{U}$ value and Young’s modulus results, it can be concluded that the anisotropy of each phase is MoC > V_2_C > VC > Cr_2_C > CrC > Mo_2_C > Fe_2_Mo_4_C > Fe_3_Mo_3_C.

### 3.3. Electronic Structure Analysis

**Figure 6:**
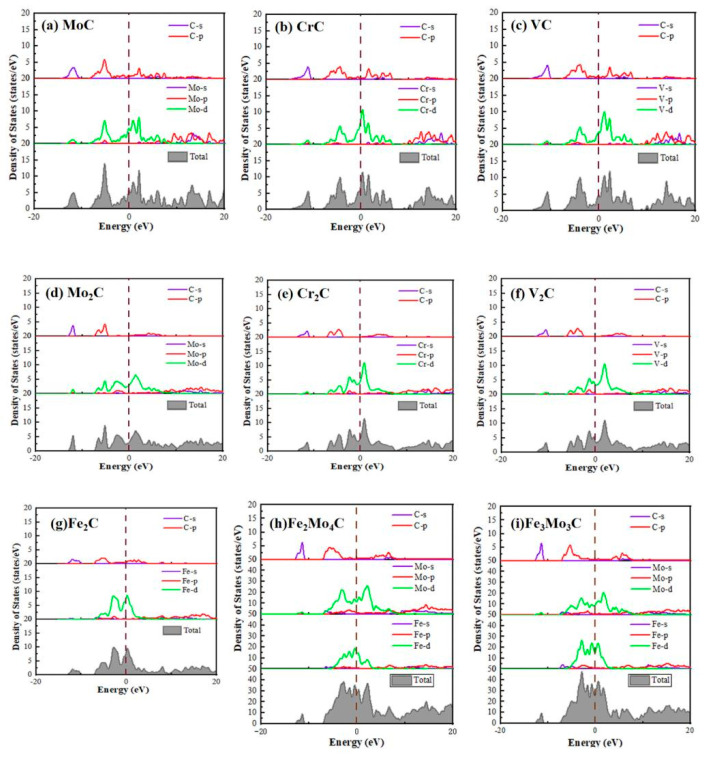
Total density of states and partial density of states diagrams of different carbides.

In order to explore the electronic properties of carbide, the density of states of carbide was calculated, as shown in Figure 6. The black vertical dashed line represents the Fermi surface. It can be seen that the valence electrons of these phases are mainly distributed from −15 eV to 20 eV. The density of states of MoC, CrC and VC are compared and analyzed. Generally, the density of states at the Fermi level indicates the number of free electrons in the bonding orbit. The lower the density of states, the more free electrons the compound has, and the more stable the structure of the corresponding compound [45]. It can be concluded from Figure 6a–c that the total density of states of CrC for the MC carbides type is the largest at the Fermi level, that is, it is more unstable, which is related to the outer electron distribution of Cr atom. This is consistent with the results of binding energy analysis, which further verify that the stability order is MoC > VC > CrC. For M_2_C (taking Mo_2_C as an example) carbides, they still have their resonance peaks generated by the C-s orbital and Mo-d orbital hybridization between −15 eV and −10 eV, which is similar to the situation of MC. In the energy range of −8 eV to 10 eV, it mainly contains the resonance peaks generated by the C-p orbital and Mo-d orbital. It is found that the stability order is Mo_2_C > V_2_C > Cr_2_C > Fe_2_C when the peak of the total density of states is compared. For M_6_C carbides, the bonding peaks are mainly contributed by C-s orbital and Mo-d orbital between −15 eV and −10 eV. The primary bonding peaks between −10 eV and 10 eV are mainly contributed to by the C-p orbital, Mo-d orbital and Fe-d orbital. For Fe_2_Mo_4_C, the peak value of the Fe-d orbital is lower than that of Fe_3_Mo_3_C, while the peak value of the Mo-d orbital is larger than that of Fe_3_Mo_3_C. Concluded from the analysis of the total density of states, the stability order is Fe_3_Mo_3_C > Fe_2_Mo_4_C. In addition, a “pseudogap” exists in all carbides. It indicates that there are covalent bonds in the crystal structure, and the width of the pseudogap can reflect the strength of the covalent bonds in the system. In general, the wider the pseudogap, the stronger the covalent bond [46]. It can be seen from the diagrams that the covalent bond strength order is MC > M_2_C > M_6_C.

**Figure 7:**
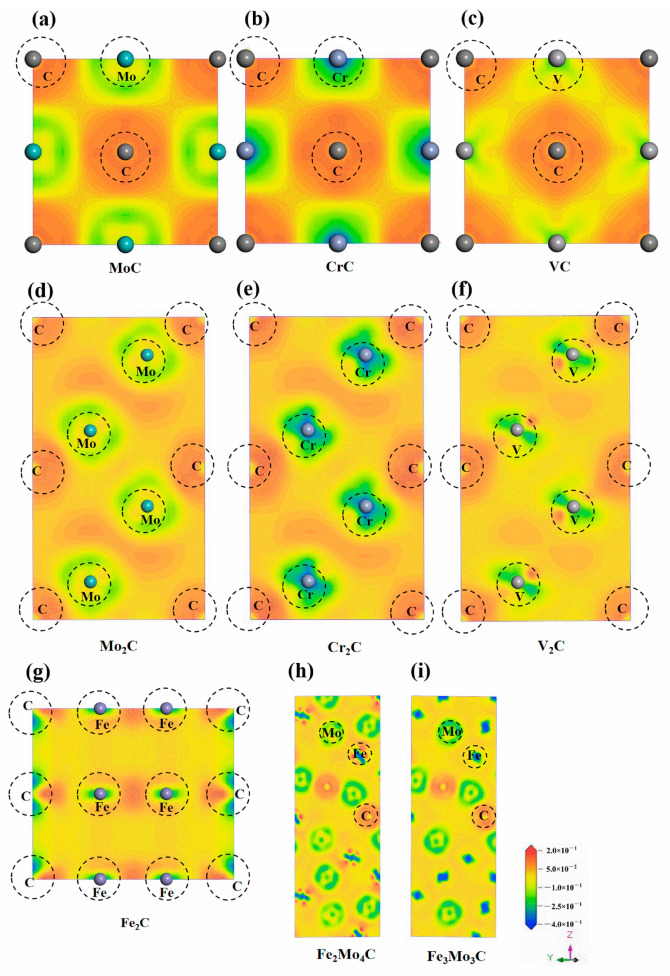
Charge density difference diagrams of different carbides. (**a**–**i**) refers to MoC, CrC, VC, Mo_2_C, Cr_2_C, V_2_C, Fe_2_C, Fe_2_Mo_4_C, Fe_3_Mo_3_C, respectively.

In order to further analyze the electronic properties of different carbides, the charge density difference in each carbide was calculated and analyzed (as shown in Figure 7). The red area represents the charge accumulation and the blue area represents the charge consumption. It can be concluded from the diagrams that there is apparent electron dispersion around the metal atom, and there is charge accumulation around the C atom. This indicates that the charge will be transferred from the metal atom to C atom, and the metal bond will form between the metal atom and C atom. For MC carbides, there are significant differences in the charge density difference diagrams. The electron dispersion around the V atom is more apparent, and the electron transfer is more obvious. It indicates that the V atom consumes more charges, and more charges are transferred to the C atom, which results in better stability. There is also a larger blue area around the Mo atom. The charge consumption around the Cr atoms is the smallest, with only a small amount of electron transfer. This is consistent with the results of previous stability analysis. For M_2_C carbides, the charge consumption around the Mo atom is the largest while the charge dispersion area of the V atom is far, and there is also charge dispersion around the Cr atom. There is an obvious and directional charge distribution in Fe_2_C, indicating that covalent bonds formed between atoms. For M_6_C carbides, the charge dispersion around the Mo atom is more obvious in Fe_2_Mo_4_C and Fe_3_Mo_3_C, while the charge dispersion around the Fe atom is smaller. The charge distribution in Fe_2_Mo_4_C is denser and its structure is more stable. The results of charge density difference and density of states are mutually demonstrated, and they are consistent with the results of binding energy analysis.

## 4. Conclusions

In this paper, the formation enthalpy, binding energy, elastic constants and general anisotropy index $A^{U}$ of typical intermediate phases in M50NiL steel were calculated using the first-principles method, and the stability, mechanical property and electronic structure of these phases are analyzed. The main conclusions are as follows:

(1) The binding energies of all phases are less than 0, indicating that these phases are stable. In the same phase, the stability of the second phase is related to the electron loss ability of its metal elements. The stronger the electron loss ability of its metal elements, the more stable the formed phase. The stability order of MC carbides is VC > MoC > CrC, while the stability order of M_2_C carbides is Mo_2_C > V_2_C > Cr_2_C > Fe_2_C. As for M_6_C carbides, the stability order is Fe_2_Mo_4_C > Fe_3_Mo_3_C.
(2) Each phase’s hardness, ductility and anisotropy were analyzed by calculating the elastic modulus and anisotropy index $A^{U}$. The hardness was measured by the Young’s modulus and the shear modulus. The greater the Young’s modulus and the shear modulus, the greater the hardness. For MC carbides, the hardness order is VC > CrC > MoC. As for M_2_C carbides, the hardness order is Mo_2_C > Cr_2_C > V_2_C > Fe_2_C. As for M_6_C carbides, the hardness order is Fe_3_Mo_3_C > Fe_2_Mo_4_C. The toughness and ductility order of all carbides is Fe_2_C > MoC > Fe_2_Mo_4_C > V_2_C > Fe_3_Mo_3_C > CrC > VC > Mo_2_C > Cr_2_C, and the anisotropy order of all carbides is MoC > V_2_C > VC > Cr_2_C > CrC > Mo_2_C > Fe_2_Mo_4_C > Fe_3_Mo_3_C.
(3) The electronic properties of all carbides are analyzed. The results of the density of states and the charge density difference show that ionic bonds and covalent bonds will form in most carbides. The main reason is that the metal atom loses electrons, and the C atom obtains electrons. The stability of all carbides also conforms to the calculation results of binding energy.

## Figures and Tables

**Table 1:** Content of each element in M50NiL-bearing steel (wt.%).

Element	Mass Percentage/%	YB/T4106-2000 [13]
C	0.147	0.13~0.15
Cr	4.14	4.00~4.25
Mo	4.28	4.00~4.50
Ni	3.37	3.20~3.60
V	1.17	1.13~1.33
Nb	0.02	-
P	0.003	≤0.008
S	0.002	≤0.003

**Table 2:** Structures and lattice parameters of different carbides.

Carbides		Space Group	Lattice System	*a (*Å)	*b* (Å)	*c* (Å)	Volume (Å^3^)
MC	MoC	$Fm\bar{3}m$	cubic	4.383	-	-	84.206
	CrC	$Fm\bar{3}m$	cubic	4.108	-	-	69.330
	VC	$Fm\bar{3}m$	cubic	4.162	-	-	72.095
M_2_C	Mo_2_C	$P\bar{3}m1$	hexagonal	3.073	3.073	4.653	38.052
	Cr_2_C	$P\bar{3}m1$	hexagonal	2.833	2.833	4.259	29.603
	V_2_C	$P\bar{3}m1$	hexagonal	2.893	2.893	4.532	32.849
	Fe_2_C	P6/mmm	hexagonal	3.699	3.699	2.642	31.307
M_6_C	Fe_2_Mo_4_C	$Fd\bar{3}m$	cubic	8.016	8.016	8.016	364.214
	Fe_3_Mo_3_C	$Fd\bar{3}m$	cubic	7.824	7.824	7.824	338.668

**Table 3:** Formation enthalpy and binding energy of different carbides.

Carbides		Enthalpy(eV)	Formation Enthalpy (eV/atom)	Binding Energy (eV/atom)	Reference
MC	MoC	−8371.20	−73.73	−9.22	−10.06 [27]
	CrC	−10,495.99	−69.46	−8.68	−9.56 [28]
	VC	−8534.52	−71.97	−9.00	−8.1 [29]
M_2_C	Mo_2_C	−8062.40	−57.37	−9.56	-
	Cr_2_C	−10,187.09	−53.00	−8.83	-
	V_2_C	−8223.31	−53.19	−8.86	-
	Fe_2_C	−3772.87	−45.46	−7.58	-
M_6_C	Fe_2_Mo_4_C	−38,566.18	−261.65	−9.34	-
	Fe_3_Mo_3_C	−34,285.21	−258.38	−9.23	-

**Table 4:** Compositions of different carbides (at.%) [35].

Carbides	Fe	Cr	Mo	V
M_6_C	35	7	56	2
M_2_C	3	13	69	15
MC	1	6	40	53

**Table 5:** Bulk modulus, Shear modulus, Young’s modulus, Poisson’s ratio, *B*/*G* and $A^{U}$ of different carbides.

Carbides		*B* (GPa)	*G* (GPa)	*E* (GPa)	ν	*B*/*G*	$A^{U}$
MC	MoC	355.97	85.86	238.42	0.39	4.15	2.87
	Ref.	328.8 [27]					
	CrC	322.24	170.32	434.41	0.28	1.89	0.26
	Ref.	317.7 [28]	156.3 [28]	402.8 [28]	0.29 [28]	2.03 [28]	
	VC	305.19	178.08	447.24	0.26	1.71	0.34
M_2_C	Mo_2_C	269.58	151.10	381.94	0.26	1.78	0.20
	Cr_2_C	248.75	146.58	367.54	0.25	1.70	0.29
	V_2_C	217.68	102.96	266.80	0.30	2.11	2.12
	Fe_2_C	198.44	25.22	72.59	0.44	7.87	-
M_6_C	Fe_2_Mo_4_C	282.98	125.58	328.20	0.31	2.25	0.001
	Fe_3_Mo_3_C	295.12	144.92	373.61	0.29	2.04	0.000

## Data Availability

The data that support the findings of this study are available on request from the authors.
